# Speciation of Genes and Genomes: Conservation of DNA Polymorphism by Barriers to Recombination Raised by Mismatch Repair System

**DOI:** 10.3389/fgene.2022.803690

**Published:** 2022-02-28

**Authors:** Miroslav Radman

**Affiliations:** ^1^ Mediterranean Institute for Life Sciences—MedILS, Split, Croatia; ^2^ Faculty of Medicine, University R. Descartes, Paris, France; ^3^ NAOS Institute for Life Sciences, Aix-en-Provence, France; ^4^ School of Medicine, University of Split, Split, Croatia

**Keywords:** mutation, recombination, mismatch repair (MMR), polymorphism, speciation, gene families, MHC, immune surveillance

## Abstract

Some basic aspects of human and animal biology and evolution involve the establishment of biological uniqueness of species and individuals within their huge variety. The discrimination among closely related species occurs in their offspring at the level of chromosomal DNA sequence homology, which is required for fertility as the hallmark of species. Biological identification of individuals, i.e., of their biological “self”, occurs at the level of protein sequences presented by the MHC/HLA complex as part of the immune system that discriminates non-self from self. Here, a mechanistic molecular model is presented that can explain how DNA sequence divergence and the activity of key mismatch repair proteins, MutS and MutL, lead to 1) genetic separation of closely related species (sympatric speciation) (Fitch and Ayala, Proceedings of the National Academy of Sciences, 1994, 91, 6717–6720), 2) the stability of genomes riddled by diverged repeated sequences, and 3) conservation of highly polymorphic DNA sequence blocks that constitute the immunological self. All three phenomena involve suppression of recombination between diverged homologies, resulting in prevention of gene sharing between closely related genomes (evolution of new species) as well as sequence sharing between closely related genes within a genome (e.g., evolution of immunoglobulin, MHC, and other gene families bearing conserved polymorphisms).

## General Introduction

Ernst Mayr defined biological species essentially by reproductive isolation of natural populations that consist of meta-lineages of sexually reproducing organisms (Queiroz, 2005). The absence of significant mixing of parental genes in the reproducing progeny would extend this species definition also to prokaryotic and other asexual species. In the latter case, sex means rare horizontal gene transfer that is facultative, i.e., unrelated to reproduction that occurs basically by clonal expansion. In closely related sexually reproducing organisms, reproductive isolation and non-mixing of genes is diagnosed after mating by the absence or sterility of the offspring. Therefore, the sterility of robust mules and hinnies testifies that their parents, horse and donkey, are different species.

Species identity is checked at the level of the DNA sequence matching of maternal and paternal chromosomes that occurs during meiosis and determines the frequency and pattern of crossovers between polymorphic parental chromosomes (Radman and Wagner, 1993). Crossovers in meiosis are mechanistically required for correct disjunction and segregation of chromosomes into haploid gametes, which are necessary for their viability. Fecundity at fertilization is diagnostic of the genomic fitness, i.e., the content of active genes in partner gametes that is decisive for fertility in sexual reproduction.

Sterility defines different species, irrespective of parental morphological and physiological similarity. For instance, we can imagine two physiologically and morphologically identical mating partners bearing genomes saturated with synonymous base substitution mutations (mostly third codon letter changes) and yet with identical proteomes. However, about 30% of densely spread sequence divergence between parental genes (even higher in non-coding genomic regions) would preclude homologous recombination. Their mating would be sterile, and their genes would not mix when altered in the future. Although morphologically and physiologically identical (with sole exception of the DNA sequence), such mating partners would not share genes and therefore be and remain different species.

However, one could easily engineer de-speciation by inserting in each of diverged parental homologous chromosome pairs, at allelic sites, chromosome-specific blocks of any arbitrary—but identical—DNA sequence of significant length. With such simple genetic engineering, the above sterile cross should become fertile. The inserted blocks of sequence identity would allow for pairing and crossover within the identical blocks of each homologous chromosome pair, i.e., a meiosis producing viable fertile gametes. Below, how physiologically neutral genomic DNA sequence divergence acts as genetic barrier and species’ biological identity is discussed.

On the other hand, the immunological “self” of each individual is checked on cell surface by presenting fragments of cellular proteins *via* the MHC/HLA complex to the receptors on T cells. A cell that presents a foreign protein—or even a cell’s own protein, but mutant or chemically modified, and hence recognized as “non-self”—is bound to be destroyed by the killer T cells (Bicknell et al., 1996). Thus, organism’s own “self” cells that produce and present foreign proteins, e.g., viral, are eventually eliminated, limiting thereby the spreading of the virus. A side effect of this benefit is that immunologically “non-self” skin or other organ’s grafts, even from siblings, are rejected by patient’s immune system. Therefore, immuno-suppressive drugs are applied after organ transplantation.

Such immunology-related genetic identities are carried and mediated by a class of highly polymorphic, but conserved, ancestral haplotypes [described as “frozen blocks” (Degli-Esposti et al., 1992)], which are the constituents of particular sequence-wise highly polymorphic gene families—the antigen-presenting MHC complex. The question is: How could such polymorphisms emerge and be maintained in the face of ongoing homologous recombination (both gene conversions and crossovers)? In the long term, frequent homologous recombination among the members of MHC gene family would tend to homogenize (especially by gene conversion), delete, or rearrange (by crossovers) such repeated polymorphic sequence blocks. A mechanistic question arises whether sequence divergence within gene families, by itself, assures their *en bloc* stability and sequence conservation and, if so, how?

**FIGURE 1:**
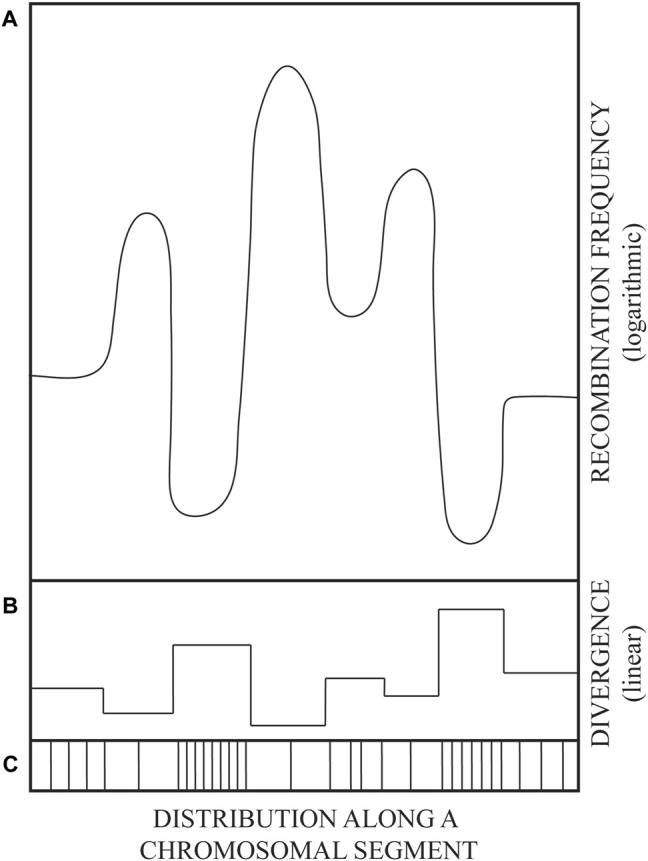
A drawing illustrating the variation in parental DNA sequence divergence (linear scale) and the corresponding variation in recombination frequencies (logarithmic scale). Schematized mirror image of DNA sequence divergence **(B)**, recombination frequency **(A)**, and the distribution of polymorphism (mutations) along an evolutionarily related genome segment **(C)**. Sketched from data in figures 4 and 5 of (Martinsohn et al., 2008). See also main text.

The observed DNA sequence divergence/polymorphism of repeated sequence blocks appears to be required for the prevention of inter-repeat homologous recombination by the activity of DNA mismatch repair (MMR) proteins (Petit et al., 1991; Vulić et al., 1997). The same kind of barriers to recombination between polymorphic sequence repeats dispersed throughout the genome can account for the maintenance of global genome integrity. Namely, if about 10^5^ LINE and 10^6^ SINE (mainly Alu) sequences, spread throughout the human genome, were identical, then the recombination among such ectopic repeats would tend to scramble the genome (Kricker et al., 1992). Likewise, recombination within burgeoning gene families would tend to shrink, or over-amplify, such repeated genetic elements by unequal, non-allelic, crossovers. Therefore, once they become biologically useful, gene families need to be stabilized. Apparently, the more diverged the repeats, the more stable they become (Figure 1). Rapid diversification seems to be required for rapid stabilization of gene families. However, how can identical repeats be rapidly diversified?

Special mechanisms of homologous interactions, other than recombination, lead to extensive methylation of cytosines (5-meC in CpG dinucleotides) with subsequent high rate of CpG→TpG transition mutations (by spontaneous or enzymatic deamination of 5-meC into T) that are targeted and limited to the length of uninterrupted homology. Homology-targeted DNA methylation is called MIP (methylation induced pre-meiotically) and RIP (repeat-induced point mutation), respectively, and were discovered initially in fungi by Eric Selker [reviewed in (Gladyshev, 2017)] but apparently occurring also in mammals (Kricker et al., 1992). Extremely high rates of mutations generated during RIP by the modification of cytosines remind of deamination of C into U during somatic hypermutation within immunoglobulin V-genes (see below).

Here, it is posited that MMR activity and DNA sequence polymorphism well-distributed throughout the genome—irrespective whether it alters the proteome—are necessary and sufficient to establish barriers to genetic recombination and maintain species’ genetic isolation and identity. The same molecular mechanism is proposed here to apply to the speciation of individual genes in emerging gene families that start with duplication of an initial “founder gene”—the common ancestor to all members of a polymorphic gene family. The immune system that consists of families of gene families is well suited for testing the proposed concepts. Personal immunological self-identity appears to be shaped by variable combinations of conserved polymorphisms of particular DNA sequence blocks in MHC/HLA regions [the “frozen blocks” of Roger Dawkins and others (Dawkins et al., 1999; Traherne et al., 2006; Dawkins, 2015; Lloyd et al., 2016)].

## DNA Sequence Variation and Conservation: Mutation *Versus* Recombination

We imagine that, during molecular evolution, there was a limited number of self-replicating ancestral RNA and/or DNA sequences that kept amplifying and diverging until naturally selected for encoding functionality. Vertical genetic variation increases sequence divergence by the accumulation of copy errors in the course of successive DNA replication rounds. Such copy errors are mainly base substitution and small insertion-deletion (or indel) mutations of one to few nucleotides. Whereas old mutations are being copied, new copy-error mutations are added and become “old” (template) mutations in the following rounds of replication and so on. This clock by which mutations are added during DNA replication allows to construct phylogenetic relatedness (trees) of genes and species by quantifying the degree of DNA sequence identity or homology.

Once a significant degree of vertical diversity is established, further genomic diversity can be generated *ad hoc* not only by horizontal variation *via* homologous genetic recombination (gene conversion and crossover) but also by occasional non-homologous small and large additions, deletions, and translocations. Recombination mechanisms between homologous sequence blocks can create in-frame or out-of-frame patchworks of pre-existing DNA sequences that are exposed to natural selection. If such successive rounds of homologous recombination were to continue beyond the early-stage partially homologous patchwork sequences, then they would lead to sequence homogenization, i.e., to a “bastard” sequence (e.g., starting by two related “black” and “white” parental sequences, continued random gene conversions that would eventually produce “grey” sequences).

Thus, the evolution of DNA sequence diversity was favored by establishing barriers to recombination. Such barriers were not raised by lowering the activity of recombination proteins, but at the level of their DNA substrate, i.e., DNA sequence polymorphism. Such solution leaves the vital process of repair of broken DNA by recombination (e.g., sister chromatid exchange) intact. Genetic recombination frequencies are tuned at substrate level by the extent and distribution of DNA sequence divergence (Figure 1), which marks the difference between “selves” in potential acts of homologous recombination.

It was demonstrated—both, in prokaryotic and eukaryotic organisms—that linear increase in random sequence divergence reduces exponentially the frequency of intra- and inter-genomic homologous recombination (Matic et al., 1996; Vulić et al., 1997; Vulić et al., 1999; Harfe and Jinks-Robertson, 2000). Such barriers to recombination between homologous but diverged (also called “homeologous”) chromosomes, as well as between repeated sequence blocks of a gene family, create genetic isolation, i.e., rare mixing of genes or gene sequences. The result is not only the stability of genomes riddled with sequence-diverged repeated DNA elements (Kricker et al., 1992) but also new speciation events (Radman and Wagner, 1993; Matic et al., 1996; Vulić et al., 1997; Vulić et al., 1999; Harfe and Jinks-Robertson, 2000).

The purpose of this paper is to propose a molecular mechanism by which the diverged, polymorphic genomic sequence blocks can be generated and then maintained as “frozen blocks” that can represent individual (e.g., tissue-type or immunological) “self” and even species “self” when such unique polymorphic “frozen” sequences persist throughout the species (Degli-Esposti et al., 1992). Predictably, conserved frozen blocks can be rearranged by recombination according to the distribution of sequence divergence, i.e., by crossovers within blocks of longest sequence identity flanking highly polymorphic frozen blocks (depicted in Figure 1).

Available data on sequence mosaicism of genes in the MHC regions—probably created by recombination—are in agreement with the principle from Figure 1: Highly polymorphic sequence blocks are themselves conserved, i.e., protected from changes within the blocks, but remain subject to rearrangements by recombination within flanking regions of lowest divergence (Legend to Figure 1 and references within). Such rearrangements, deletions (Petit et al., 1991; Elez et al., 2007), inversions (Martinsohn et al., 2008), or insertions (Shen and Huang, 1986) *via* crossovers within partially homologous sequences have been shown in model genetic constructs, both chromosomal and extrachromosomal: Crossovers occur most frequently within blocks of longest sequence identity.

Below, a molecular mechanism of the evolution of genetic diversification and, in particular, the subsequent DNA sequence stabilization and conservation establishing thereby the uniqueness and genetic identity of individuals and species is proposed.

### Physical Matching of Similar DNA Sequences During Homologous Recombination

Physical matching of identical and similar DNA sequences occurs by the cross-hybridization of complementary DNA strands from two DNA duplexes. Beyond a minimal length, reducing the length of sequence identity reduces the frequency of recombination linearly (Shen and Huang, 1986), whereas reducing homology by sequence divergence reduces recombination exponentially (Vulić et al., 1997). Finding and hybridizing identical and similar sequences is the key step in homologous genetic recombination. Number, distribution, and nature of mismatched base pairs in the hybrid or heteroduplex DNA characterize DNA sequence divergence.

**FIGURE 2:**
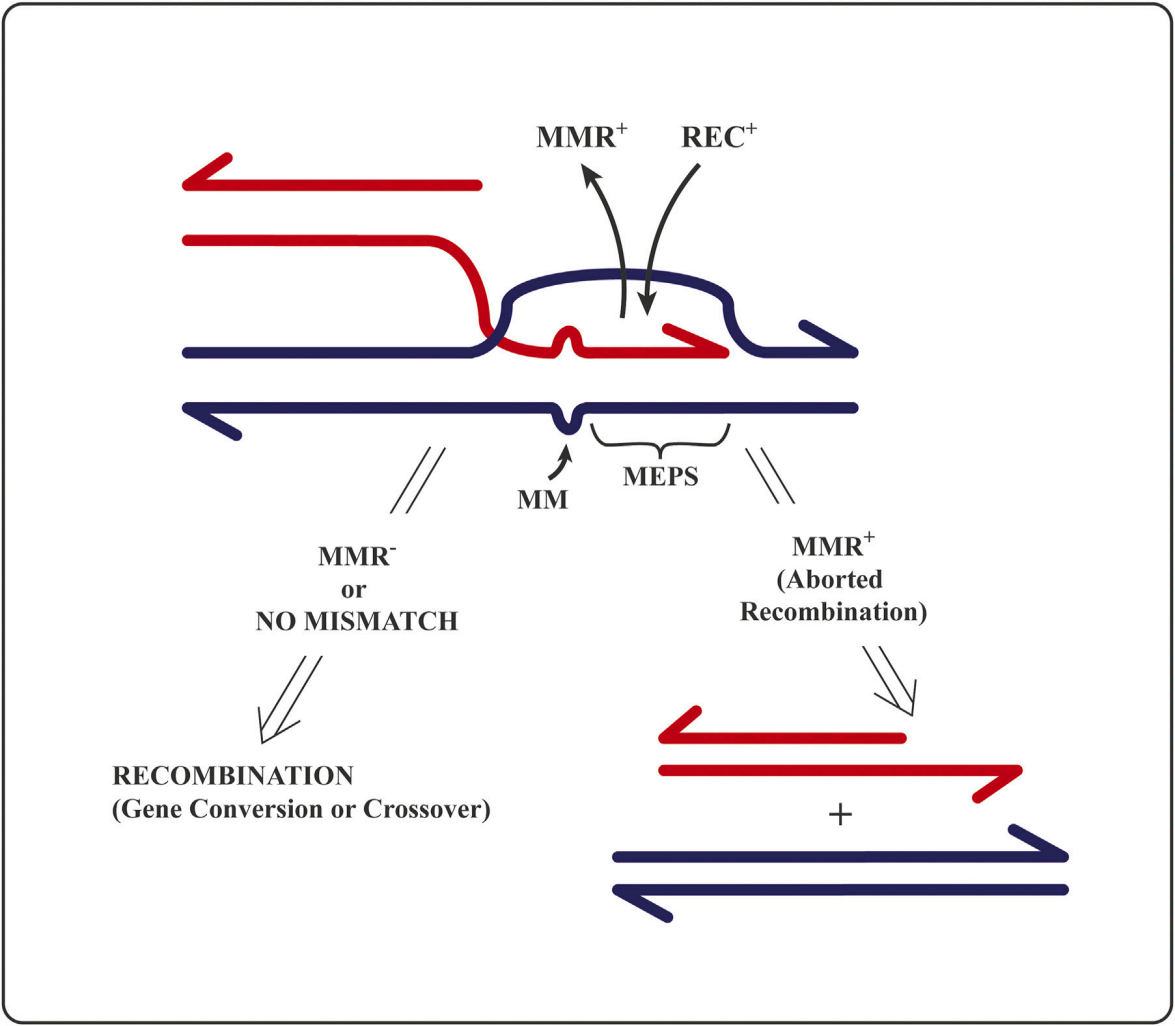
A drawing of the mechanistic principle for the editing of homologous recombination by mismatch repair proteins: Reversal of an attempt to initiate homologous recombination between non-identical (homeologous) sequences. *MMR* ^***+***^ stands for mismatch recognition and repair proteins; *REC* ^***+***^ for recombination proteins at the DNA strand invasion step; *MM* are mismatched or unmatched bases; *MEPS* is “minimal efficient processing segment”, i.e., minimal sufficient block of strict sequence identity required to initiate strand exchange (ref 13). RecBCD nuclease is known to degrade the free-ended (red) DNA that did not succeed in recombination (Štambuk and Radman, 1998) precluding repeated recombination attempts at the already edited site. See text and refs. (Shen and Huang, 1986; Radman, 1989; Kowalczykowski et al., 1994; Worth et al., 1994; Štambuk and Radman, 1998; Wiktor et al., 2021).

To initiate homologous recombination, the recombination machinery (protein complex associated with the prototypic homolog of the bacterial RecA recombinase) requires a minimum length of strict sequence identity, called MEPS (for minimal efficient processing segment) (Shen and Huang, 1986). MEPS corresponds to the length of single-stranded DNA that—as RecA nucleofilament—searches the nucleus (or bacterial nucleoid) for duplex DNA stretch of identical sequence to initiate strand exchange (Wiktor et al., 2021). Bacterial MEPS is a matching sequence of, at most, 30 identical base pairs (Shen and Huang, 1986), whereas eukaryotic MEPS length is in the range between 200 (in lower eukaryotes) and 400 (in mammals) nucleotides (Kricker et al., 1992; Kowalczykowski et al., 1994; Štambuk and Radman, 1998). A block of sequence identity larger than MEPS flanked by heterology will recombine at higher frequency than when flanked by similar sequences, even of low divergence (Shen and Huang, 1986; Elez et al., 2007; Martinsohn et al., 2008). This surprising observation is explained below in terms of abortion of recombination-initiating strand exchange within identity and elongating into the region of sequence divergence, hence generating mismatched base pairs (Figure 2).

The initial molecular “kissing” between two DNA MEPSs (Figure 2) is a prerequisite to initiate a progressive strand exchange between two homologous partner DNA sequences to elongate the heteroduplex region up to several kilo-bases [reviewed in (Kricker et al., 1992)]. A heteroduplex is a DNA region of swapped strands in which one complementary DNA strand is contributed by another homologous DNA sequence (Figure 2), either as part of a homologous chromosome (allelic) or as a non-allelic, ectopic repeat.

A long heteroduplex region assures correct, allelic, reciprocal “splice” (crossover), or non-reciprocal “patch” (gene conversion) events. The symmetrical strand-exchanging cruciform-like structure, called Holliday junction, is eventually processed by resolvases that terminate the recombination event either as reciprocal (crossover) or non-reciprocal (gene conversion) event [(Kowalczykowski et al., 1994) and reviews]. In homologous recombination between non-allelic blocks, only crossovers can generate DNA rearrangements.

### Role of Mismatch Repair Proteins in Suppressing Recombination of Diverged Homologies

Although MMR system is usually, and correctly, described as the copy-error editor of DNA replication, it is of no lesser importance as the editor of homologous recombination (Jones et al., 1987; Radman, 1989; Worth et al., 1994; Štambuk and Radman, 1998; Elez et al., 2007). Loss of MutS or MutL (or their eukaryotic MSH and MLH/PMS homologs, respectively) function leads to a promiscuous homologous recombination that tolerates significant levels of sequence divergence (up to 20%). Such recombinational promiscuity has major biological consequences (Jones et al., 1987; Rayssiguier et al., 1989; Radman and Wagner, 1993).

A functional MMR system aborts (by DNA helicase dedicated to MMR) the attempts to initiate and/or to extend strand exchange in recombination as soon as single–base pair mismatches are created at sites of sequence non-identity within the hybrid DNA region. Hybrid or heteroduplex region is formed by the exchange (swapping) of DNA strands of the same sequence and polarity belonging to two parental DNA sequences (Figure 2). The bacterial MutS homodimer protein (and its eukaryotic heterodimer homologs) evolved to diagnose (recognize and bind to) single–DNA base pair mismatches and small indel (one to three bases) non-matches in heteroduplex DNA. Only then, MutL (and its eukaryotic heterodimer homologs) bind to the mismatch-bound MutS and attract a dedicated helicase (Jones et al., 1987; Radman, 1989; Worth et al., 1994; Štambuk and Radman, 1998; Elez et al., 2007), which will undo the heteroduplex DNA generated by recombination proteins (Figure 2). Hence, the initiated recombination event will be reversed/aborted, and the two DNA molecules partnering in recombination will be separated. Such prevention of attempted recombination between non-identical partner sequences creates barriers to recombination (Figures 1, 2).

The observation that, in the absence of active MutS and MutL proteins, shorter than normal MEPSs can initiate recombination (Štambuk and Radman, 1998) suggests the association of recombination-editing MMR proteins with the recombination machinery. Such association would assure early detection of sequence non-identity (as mismatched base pairs in heteroduplex DNA), stop heteroduplex extension, and abort the initiated recombination process (Jones et al., 1987; Rayssiguier et al., 1989; Worth et al., 1994; Štambuk and Radman, 1998; Elez et al., 2007). Long heteroduplex DNA in homologous recombination allows to MMR proteins to spot sequence non-identity by detecting individual DNA base pair mismatches and then actively prevent recombination among sequences that are not strictly identical (Figure 2). Homologous recombination between identical sequences is apparently irreversible, whereas recombination between non-identical sequences is apparently mostly reversible (Worth et al., 1994). This is the general principle of the kinetic proofreading theory (off/on rate ratio is increased for wrong substrates and intermediates in biosynthetic processes). Such editorial proofreading of homologous genetic recombination based on single mismatch recognition by MMR system is so efficient that the interruptions of sequence identity by base substitution and small indel mutations is much more effective in preventing recombination than the interruptions of sequence identity by large insertions and deletions within identical sequences (Matic et al., 1996). Such large heterologies either stop the extension of strand exchange and mature the recombinant structure by resolvases or form large heteroduplex loops that are not recognized or processed by the MMR system (Dohet et al., 1987).

### Genetics and Biology of Barriers to Recombination

Homologous recombination is so sensitive to base pair mismatches that even single mismatches (e.g., due to mutations used as markers in genetic crosses) affect genetic recombination frequency, especially when closely spaced. For example, the use of closely linked genetic markers can modify genetic recombination frequencies in MMR proficient cells by several orders of magnitude (Calos and Miller, 1981; Jones et al., 1987). Thus, the mutational nature of genetic markers and the efficiency of mismatch recognition by MMR proteins introduce an uncertainty principle in fine genetic mapping using closely spaced mutations.

With this in mind, an inter-strain or inter-species genetic cross can be considered as a hundred or million factor (i.e., mutational marker) cross, involving phenotypically silent mutations that raise barriers to recombination (Rayssiguier et al., 1989; de Wind et al., 1995; Hunter et al., 1996).

In reality, only sister chromatids issued from the replication of a single “mother” DNA molecule are identical (short of rare newly arising mutations) and thus allowed to freely recombine to repair DNA damage, e.g., replication-associated double-strand breaks in DNA. Any mutation present in the copied intact DNA partner molecule will be transferred into DSB or gap region repaired by homologous recombination. It is called gene conversion, and it is the principle of genome editing for correcting disease-causing mutations. Double-stranded gaps in DNA can be repaired only by retrieving the missing sequence from another identical intact DNA sequence *via* the synthesis of the missing sequence on an intact homologous template. Such information-retrieving process is homologous recombination that requires two DNA sequences interacting *via* recombination and replication proteins.

Sister chromatid exchanges are inter-chromatid crossovers that are quite frequent (several in each mitotic cycle of mammalian cells) yet genetically inconsequential when precisely allelic. However, mitotic recombination between homologous “mom” and “dad” chromosomes in diploid somatic cells is rare because it is prevented by the mom-dad genomic sequence divergence [i.e., polymorphism by single-nucleotide variation (SNV)], which is about 0.1% in human population.

**FIGURE 3:**
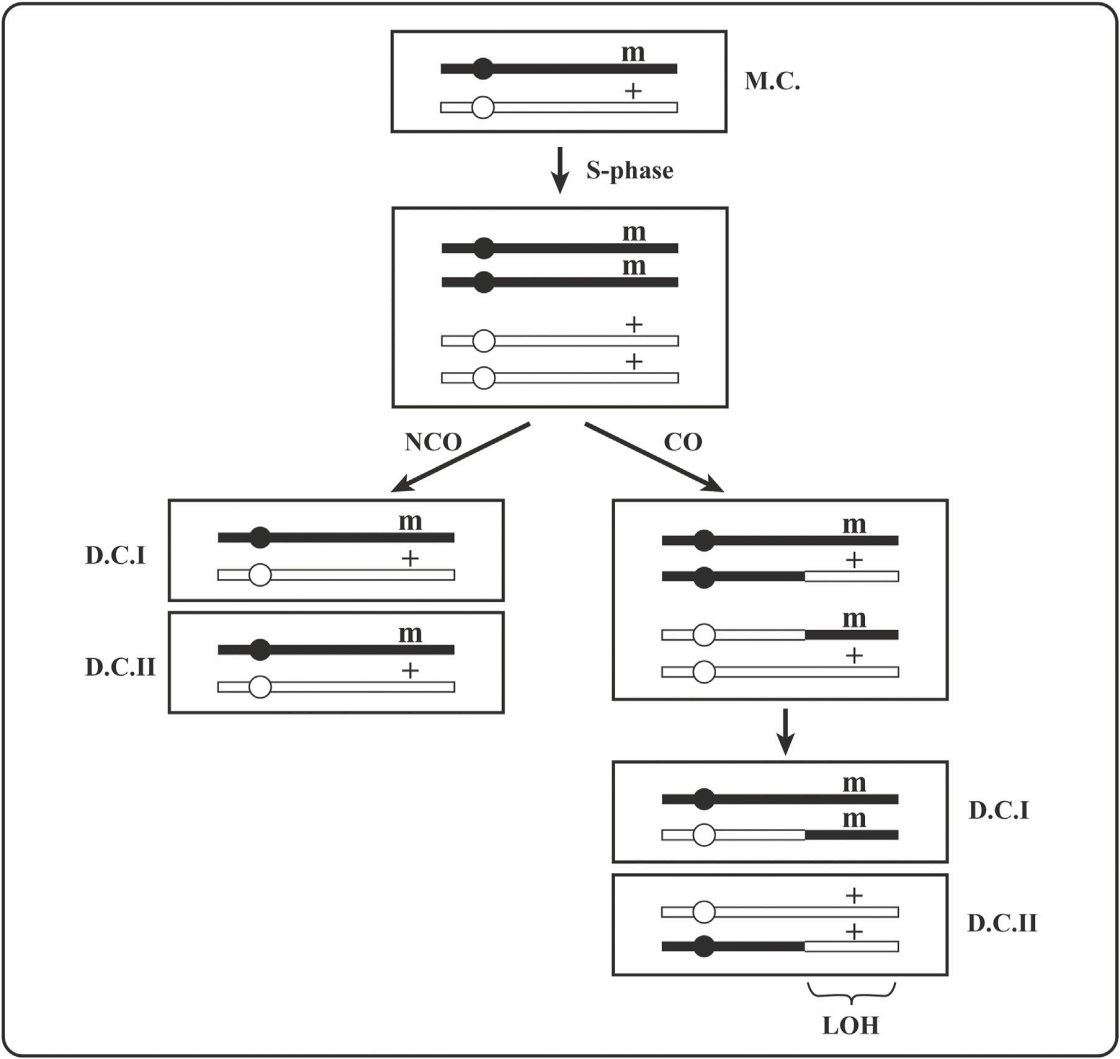
A drawing of the expression of a recessive heterozygous mutation by its segregation to homozygosity *via* mitotic recombination in mother cell. **(+)** is an active gene, **(m)** is its inactive mutant version; **MC** is a heterozygous (+/m) mother cell and **DC** are heterozygous (+/m) or homozygous (+/+ and m/m) daughter cells; **CO** stands for crossover and **NCO** for non-crossover. Gene conversion that can create +/+ or m/m homozygosity is not shown. **LOH** is loss of heterozygosity for all parental (maternal or paternal) markers (SNPs) extending from the crossover site to the end of the chromosome.

The suppression of mitotic recombination is precious because it prevents expression of recessive, heterozygous, phenotypically silent loss-of-function mutations or epigenetic gene silencing events. Two daughter cells issued from division of a heterozygous (+/m) mother cell become homozygous (one as m/m and the other as +/+) by mitotic crossover (Figure 3) or by gene conversion of (+) locus to (m). Because a crossover anywhere between the centromere and the (+/m) locus will lead to (+/+) and (m/m) homozygosity (Figure 1), it is more likely to lead to homozygosity than gene conversion or new mutation in that locus. Moreover, homozygosity acquired by crossover extends as loss of heterozygosity (LOH) for all SNV from the crossover site to the end of the chromosome (Figure 3).

The expression of phenotypically silent recessive heterozygous mutations (+/m) is a necessary step in carcinogenesis [e.g., for the loss of tumor suppressor function in (m/m) cells, as in Figure 3]. The consequences of mitotic recombination are observed massively in carcinomas as LOH affecting significant to large fraction of tumor cell genome (e.g., often over 50% of the genome in cells of some melanomas). Normally, genome-wide heterozygosity (involving mostly SNPs) of diploid cells is due to non-identity of mom and dad’s chromosomal sequences that will become homozygous (mom-only or dad-only) by mitotic crossovers, as depicted in Figure 3.

The expression of a phenotypically silent heterozygous recessive mutation (m/+ in Figure 3) *via* its homozygosity (m/m) by mitotic recombination was shown in heterozygous APRT +/m mice to be up to 80 times more frequent than by all other events together [e.g., a second APRT mutation (+→m), deletions that include (+/m) locus and chromosome loss (hemizygosity), or epigenetic APRT gene silencing]. However, this result holds only for inbred mice where mom and dad chromosomes are nearly identical (Shao et al., 1999; Shao et al., 2001). In mice that are hybrids of two different strains, i.e., similar to “wild” mice in terms of SNP heterozygosity, the genome-wide polymorphism reduces the frequency of mitotic recombination as to bring it down to the level of gene mutation frequency (Shao et al., 1999; Shao et al., 2001).

As expected, when deficient in MMR, both inbred and “wild” (hybrid) mice display similar high frequencies of cellular homozygosity (APRT m/m) by mitotic recombination in tissues bearing the recessive (APRT+/m) mutation (Shao et al., 1999; Shao et al., 2001). Thus, by suppressing mitotic recombination, MMR system prevents the expression of heterozygous recessive mutations (m/+) when homologous chromosomes are polymorphic (non-identical), as is the case of individuals born to unrelated parents. An exaggeration of parental un-relatedness is when parents belong to different species. For example, inbred horses tend to get cancers more frequently than the non-inbred horses, in particular, when compared to their health-wise robust but sterile cousins, mules and hinnies, bearing highly diverged parental (horse–donkey) genomes (as per the interviews with over 20 veterinarians). Although the interpretation of these observations is uncertain, it just might be that the disease avoidance and reproductive sterility can be the pronounced phenotypic consequences—mitotic and meiotic, respectively—of otherwise silent genomic DNA polymorphisms that creates the genomic identity of individuals and species.

The power of natural low-level genomic polymorphism in preventing homologous recombination was demonstrated by a 4000-fold lower efficiency of experimental gene replacement *via* homologous recombination in mouse embryonic stem (ES) cells when using non-isogenic *versus* isogenic DNA (de Wind et al., 1995). Apparently, functionally silent DNA sequence divergence (within the Rb gene-replacing DNA block) between mouse donor and recipient strains is the cause of the suppression of homologous gene replacement (de Wind et al., 1995) because this large difference between inbred isogenic and non-isogenic gene replacement frequencies vanishes in MMR deficient (MSH2−/−) ES cells. In both cases, gene replacement occurs at equal, high frequency (de Wind et al., 1995). This will eventually become relevant to the efficiency and precision of gene therapy by DNA sequence replacement because all current approaches depend on homologous recombination in living cells.

### Speciation by Barriers to Recombination

Speciation is the process of emergence of new species as the appearance of lineages that are reproductively and genetically isolated from other closely related lineages. Inter-species recombination between co-linear (syntenic) genomes (i.e., the genomes with conserved gene order) of the bacterial genera *Escherichia* and *Salmonella*—with about 20% sequence divergence by well-spread, mainly silent, synonymous mutations—is reduced by at least 10^4^-fold compared to intra-species recombination (Rayssiguier et al., 1989; Matic et al., 2000). However, when bacterial “female” (F) cell that receives DNA from a “male” Hfr donor cell is deficient in MutS or MutL MMR function, then inter-species recombination frequency approaches that of intra-species recombination (Rayssiguier et al., 1989; Vulić et al., 1997; Matic et al., 2000). Thus, formally, MMR deficiency triggers “de-speciation”. Apparently, genomic sequence polymorphism and MMR activity constitute the genetic barrier between closely related species, including eukaryotic species (see below).

That MMR activity and genomic DNA sequence divergence/polymorphism are sufficient to constitute inter-species genetic barrier in bacterial speciation was demonstrated by a first-time experimental incipient speciation in the laboratory—in real time (Vulić et al., 1999). Richard Lenski and colleagues started an ongoing, several decades-long, growth of *Escherchia coli* from a single colony grown by successive divisions of an initial single bacterial cell. Twelve parallel cultures were inoculated and allowed to grow by daily dilutions with sterile growth medium for over 20,000 cell generations (in 1997, at the time of our experiment) while keeping the historical samples frozen. At different times, four out of 12 cultures were overtaken by spontaneously emerging MutS or MutL deficient *E. coli* mutator mutants because mutators generate adaptive mutations—and about 10,000 times more frequent neutral and deleterious mutations (Taddei et al., 1997)—at about 100 times higher rate than the initial wild-type ancestor. During over 20,000 cell generations in Lenski’s laboratory, mutator bacteria rapidly accumulated sufficient genomic polymorphisms (about 0.03%–0.06% in 1997) as to reduce recombination, by 10-fold, in crosses with the conserved ancestor clone—or with a parallel mutator culture with doubled divergence and further reduced recombination (Vulić et al., 1999). However, this reduction in recombination was fully MMR dependent, i.e., seen only once the MutS/L proficiency was restored! Thus, phenotypically neutral genomic sequence divergence, which had accumulated during continuous growth in the laboratory, reduced the recombination with the ancestor cell in a MutS/L-dependent fashion. Today, after over 60,000 generations, the barrier to recombination would expectedly be more impressive. This is an experimental model for (bacterial) speciation occurring in real time over decades of continuous sympatric clonal growth in the laboratory (Vulić et al., 1999).

Inspired by experiments with bacteria (Rayssiguier et al., 1989), Rhona Borts and others demonstrated in the crosses between two closely related yeast species that the barriers to meiotic recombination—and the consequent sterility, i.e., the non-viability of haploid spores—are due to genomic sequence polymorphism and MMR activity (Hunter et al., 1996). They performed basically sterile inter-species crosses between two about of 10% diverged largely syntenic yeast of the *Saccharomyces* genus (*S. cerevisiae* and *S. paradoxus*). Although the haploid spores of two budding yeast species fuse and grow as normal diploid cells, the spores generated after the meiotic divisions of the hybrid diploid are inviable due to aneuploidy caused by the lack of crossing-overs between inter-specific chromosomes (Hunter et al., 1996). Such yeast sterility, like that of mules and hinnies, defines that the two yeast unicellular sexual partners belong to different species even if they are physiologically nearly identical, e.g., diverged largely by synonymous mutations that do not alter amino acids in proteins.

The sterility of genetic crosses is key to the operational definition of species by Ernst Meyr (Queiroz, 2005), meaning that there is reproductive isolation and the absence of gene sharing. Knocking-out the MSH2 gene in both haploid yeast cell partners of two species (before their fusion) generated a MMR-deficient diploid cell that upon induced meiosis and sporulation gave rise up to 30% of viable haploid spores containing a complete set of chromosomes that have all undergone homologous inter-specific crossing overs (Hunter et al., 1996). Therefore, each chromosome from such viable spores was a different interspecies mosaic. Hence, by the formal definition of species, each viable product (spore) of these inter-species crosses—fertile only when MMR deficient—is a new yeast species under the normal wild-type MMR regimen.

A conclusion of this chapter is that, in the crosses between closely related species, the MMR status decides when they are the same and when they are a different species. With non-functional MMR, they are the same species; with functional MMR, they are different species. Overexpression of MMR proteins further reduces bacterial interspecies recombination and formally leads to an *ad hoc* speciation (Vulić et al., 1997; Vulić et al., 1999; Denamur et al., 2000), whereas MMR loss leads to *ad hoc* “de-speciation” (Denamur et al., 2000). Hence, the references (Vulić et al., 1997; Vulić et al., 1999; Denamur et al., 2000) support a vision of sympatric speciation by punctuated equilibrium (i.e., bursts of speciation) by reiteration of the loss and regain of MMR that acts *de facto* as a “speciator” (Denamur et al., 2000). The 100-fold mutator effect of the loss of MMR accelerates the generation of polymorphism but also lowers genetic (recombinational) barriers. Regain of MMR lost by mutation *via* well-documented frequent, or strongly selected, horizontal gene transfer of MMR gene sequences in the evolutionary past (Denamur et al., 2000) acts upon the acquired DNA sequence polymorphism to rise, *ad hoc*, new genetic barriers that produce bursts of sympatric speciation akin to punctuated equilibrium (Vulić et al., 1997; Vulić et al., 1999; Denamur et al., 2000).

### “Speciation” of Genes Within Gene Families: Genetic Isolation of Related Genes and the Polymorphism of “Frozen Blocks”

Gene duplication events produce first an identical tandem gene pair that can readily expand and/or shrink in number by unequal crossovers between sister chromatids, get stabilized, and eventually become a gene family. As soon as first mutation occurs in one of the gene copies, MMR will reduce its recombination with other members of the gene family. Later on, mutations will keep accumulating in all of the gene copies, further reducing recombination among them, and, hence, conserve the accumulating polymorphism. When members of such gene family are not usefully expressed, they will drift away from the initial sequence by accumulating mutations and finally become either inactive pseudogenes or genes acquiring new functions.

However, when the very function of such (pseudo)gene family consists of rapid, almost instant, generation of diversity, such as in the case of the variable (V) region of immunoglobulin genes or of special T-cell receptors, then the genetic capital of that gene family corresponds to the wealth of actual, as well as potential, diversity that can be generated by gene conversions among individual members of gene family. After VDJ rearrangement, the antibody maturation (i.e., increase in the affinity for the antigen) occurs within hours by V-gene hyper-variability. Such hyper-variability is triggered by the activation (*via* antigen binding) of either hyper-conversion with other silent V-pseudogenes [as in chicken and rabbit, (Reynaud et al., 1987; Becker and Knight, 1990)] or targeted hyper-mutation by AID (activation-induced deamination of cytosine into uracil), as in mouse and man (Petersen-Mahrt et al., 2002). Both mechanisms appear to be activated by the AID activity possibly by DNA breakage in the expressed V-gene caused by the repair (by uracil N-glycosylase) of closely spaced uracils produced by AID in transiently single-stranded DNA during transcription (Arakawa et al., 2002).

Hyper-conversion generates immunoglobulin maturation *via* V-gene variability by creating sequence patchworks/mosaics within the transcribed V-gene using chunks of sequences from silent (non-transcribed) V-pseudogenes (Reynaud et al., 1987; Becker and Knight, 1990). Therefore, amazingly, sequences of unexpressed V-pseudogenes are kept under selective pressure when transferred by gene conversion into the expressed V-gene to generate the immunoglobulin G (IgG) repertoire in chicken and rabbits (Reynaud et al., 1987; Becker and Knight, 1990). Therefore, when different IgG V-sequences are lined up vertically, the distribution (location and density) of sequence hyper-variation in expressed V-genes resemble very much to the diversity among unexpressed V-pseudogenes (Radman, 1983) whether generated by hyper-conversion (in chicken and rabbits) or by hyper-mutation (human and mice). Apparently, the selection for antibody function, i.e., antigen binding, brings out the pattern of V gene hyper-variability independently of the mechanism of sequence variation.

Thus, with hyper-conversion mechanism, the selected mutational pattern became encrypted in the germ line repertoire as a sort of memory of the past evolutionary experience. Namely, the non-transcribed V-pseudogenes will transfer fragments of their inert sequence to the expressed V-gene by gene conversion and contribute functional specificities from the memory repertoire encrypted in silent pseudogenes. It turns out that both mechanisms of hyper-variation of the expressed V gene sequences coexist in mice and men and that both are triggered by AID catalyzed C→U transitions and their repair (Hunter et al., 1996). What a fascinating piece of somatic molecular genetics!

### Homologous DNA Interactions Other Than Recombination, Defense Against Genetic Parasites, and the Structure of Eukaryotic Genes

One of the troubles met in the early times of biotechnology was a progressive *trans*-generational loss of expression of artificially introduced transgenes (from the same or different species) in fungi, plants, and animals. The speed of gene silencing was related to the transgene copy number in the genome and occurred in haploid nuclei, pre-meiotically in fungi and post-fertilization, at dikaryon stage, in animals [reviewed in (Kricker et al., 1992; Gladyshev, 2017)]. Such repeat-associated gene silencing and sequence degeneration by hyper-mutation are reminiscent of MIP and RIP phenomena described above. Both require similar MEPS length (block of sequence identity) as does recombination. Such checking, in haploid nuclei, for intra-genomic sequence homology larger than about 0.3–0.4 kb looks like a diagnostic search for the most recent, identical, genomic parasites, such as viruses, retro-elements, or pseudogenes. The latter are usually fully, sometimes partially, processed before being retro-transcribed from cellular m-RNAs into c-DNA. The tendency to insert multiple copies into the genome is characteristic of genetic parasites.

To diagnose invading genetic parasites by their copy number, methylating (MIP) and then mutating (RIP) them to their genetic doom is a cunning strategy—short of the danger of deadly silencing organism’s own vital genes due to ectopic homologies of their pseudogenes. Gene silencing *via* some kind of homologous interactions of genes with their pseudogenes has been observed with c-DNA transgenes in fungi and plants when the gene was intron-less or had a single short intron [reviewed in (Kricker et al., 1992)].

When the transfection with transgene was used to amplify an authentic host gene, then—instead of expected increased gene activity by the gene dosage effect—transgene copies and the resident host gene were inactivated in Petunia plants [reviewed in (Kricker et al., 1992; Gladyshev, 2017)]. In human genome, there are more pseudogene sequences present as DNA copies of processed m-RNA from resident genes than genes themselves. Some human genes have over 200 pseudogenes (Kricker et al., 1992), constituting an awesome selective pressure on expression of homologous resident genes. A conflict of two fatalities enters: either to go extinct by massive insertions of active genetic parasites or to die from the silencing of vital cellular genes (as a side effect of the defense against extinction) because the strategy uses homology-based gene silencing by MIP and RIP kind of mechanisms.

Metazoan gene structure provides the solution to the two lethal conflicts (Kricker et al., 1992): Chop genes into short exons by insertion of long introns such that only retro-transcribed unprocessed hn-RNA would be harmful. Indeed, the length of nearly all translated exons of human genes peaks in the range of 80–120 base pairs. At first, RNA splicing may appear as a complication, but, in this constellation, it appears as a life saver by allowing MIP/RIP strategy for the silencing of genetic parasites while sparing cell’s own genes from silencing by their pseudogenes! Indeed, whenever a pseudogene contained a non-spliced intron sequence, extending the uninterrupted homology with the gene well beyond the MEPS length, the exons flanking the non-spliced intron suffered the deficit of CpG dinucleotides with equal excess of TpG—the hallmark of cytosine methylation-mediated mutagenesis (Kricker et al., 1992).

The acquisition of resistance of authentic cellular genes to epigenetic silencing by MIP and to mutational inactivation by RIP is so far the only concept of a direct selective pressure for the fragmentation of genes into short exons. Other popular ideas about the benefits of short exons are anticipatory (e.g., fragmentation of genes would facilitate shuffling of exons encoding protein modules, which would unleash the evolution of protein diversity). This benefit can happen but only once the genes are already fragmented into short exons *via* some other direct strong selective pressure to do so, as the one proposed by (Kricker et al., 1992).

This dramatic scenario of invasions of genetic parasites in the evolutionary past left a massive testimony in our germ line genome: Over one half of human genome is the cemetery of genetic parasites, mostly those retro-copied from RNA into DNA and then integrated into the genome (makes one think of mRNA and DNA vaccines!). There are about 10^5^ of LINEs elements in the human genome, which are complete or partial DNA copies of RNA retroviruses. All LINEs are found to be heterogeneously methylated on cytosine in CpG dinucleotides and massively mutated by CpG→TpG transitions [as to undergo deficit of CpG in favor of TpG, dinucleotides (Kricker et al., 1992)] such that LINEs usually, but not always, “rest in peace” by RIP-like mutagenesis.

## Summing up and Perspectives

Here, a description of basic molecular mechanisms playing part in the evolutionary strategies for the emergence and safeguard of biological integrity (functionality) and identity of species and individuals is presented. Variability from an original DNA sequence, both vertical (by point mutation) and horizontal (by recombination), generates sequence divergence, or polymorphism, which appears as the key structural element in the emergence of new species and of new gene families within the genome. The key functional element in genetic isolation (i.e., prevention of DNA sequence mixing by preventing homologous recombination) is the proteins of the MMR system, whereas sequence polymorphism is the structural element of genetic isolation of genomes and genes. Mismatched base pairs within heteroduplex DNA formed during homologous recombination and the anti-recombination activity of MMR lead to the conservation of emerging DNA sequence diversity.

Genome-wide DNA sequence polymorphism, even if functionally silent, can lead to new biological species *via* genetic isolation, i.e., prevention of gene sharing with closely related species. Hence, by editing meiotic genetic recombination, MMR system appears as a “speciator”. Even individual genes can “speciate” within their gene families by the prevention of sequence sharing *via* recombination with other members of that gene family. Such is the case of genetic structure and function of the immune system that involves discrimination between self and non-self at the level of protein sequences.

It is unlikely that there was a direct selective pressure for evolving genetic recombination and its editing by MMR to play this kind of exotic biological roles like the “selfness” of individuals and species that could difficultly be anticipated or directly selected for. More likely, there was direct selective pressure for survival *via* recombination repair of ongoing DNA breakage and *via* reduction of mutation rate by MMR. Then, later in evolution, the processes were there to be used also for other purposes (see below).

Biologists are often eager to trash sound concepts by finding “exceptions”—usually some specific observation, or localized phenomenon, grafted upon general principles. Hence, the particular mechanistic model schematized in Figure 2 is meant to serve as a general concept and mechanism for the conservation of diversity. Exceptions that can locally hide, or override, the general rule are expected in evolution: It suffices that there be a selective pressure for the emergence of “exceptions”. For instance, here, the appearance of meiotic recombinational hot spots (Jeffreys et al., 2004) as sites of targeted [e.g., by PRDM9 protein, (Wells et al., 2020)] double-strand breakage by SPO-11 topoisomerase, along with an epigenetic control of the hot-spot activity, e.g., by histone modifications.

Finally, one can appreciate the dynamics and extent of past and ongoing genetic diversification—*via* sequence variation and rearrangements of conserved polymorphic blocks occurring within the chromosomal MHC region—by overviewing the results of extensive sequencing. A single gene CYP-21 encoding 21-hydrolase (one of the “sanitation proteins”) located within the MHC region showed cases of all mechanisms discussed in this paper (Simonetti et al., 2018): new mutations, rearrangements, transfer of mutations from CYP-21 pseudogene into CYP-21 gene by gene conversion, and even loss of gene activity by a newly arising whole gene duplication event (presumably followed by MIP-like gene silencing).

If nothing essential was missed or overlooked here, then it appears that the mechanisms of emergence and maintenance of individual self-identity, as well as species’ identity, might have evolved “collaterally” under the selective pressure to limit the spreading of diverse parasitic genetic elements such as viruses and transposons. “Collaterally” refers to collateral benefit of making use of already evolved mechanisms of genetic robustness, such as MMR and recombination repair systems, under the selective pressure to survive invasions of biological “non-self” entities. Discriminating against all “non-self” appears simpler than discriminating specifically against each one of numerous biological and genetic parasites, and even small toxins. However, the proviso is that “self”/“non-self” discrimination must be flawless—best by the uniqueness of “self”.

The individual biological identity appears now as probable byproduct of the evolution of an efficient discrimination against most, or all, non-self proteins, i.e., cells that produce non-self proteins and present their small fragments *via* MHC complex. Not only non-self proteins (in infectious diseases) but also genuine “self” proteins can be foreign, which have acquired a “non-self” status by structural alterations caused by mutations or chemical damage (in age-related diseases and aging) that modify the pattern of proteolytic processing and therefore the identity of protein fragments displayed by MHC complex. Such scenario plays well with numerous associations of particular polymorphic haplotypes of the individual immune system with a plethora of infectious and non-infectious diseases, inflammation, and aging (Dawkins et al., 1999; Traherne et al., 2006; Steele, 2014; Dawkins, 2015; Lloyd et al., 2016). Such associations hint to a role of immunity in the elimination of abnormal (diseased or senescent) cells acting as a tissue clean-up system that prevents diseases and delay aging.

## An Epilogue on Biological Individuality and Diseases

It seems clear why should self/non-self discrimination *via* MHC/HLA antigen-presentation system of foreign (e.g., viral or bacterial) proteins on cell surface be involved in the defense against infectious diseases, but the association of particular MHC/HLA sequence variants—the self haplotypes—with a variety of non-infectious age-related diseases [reviewed in (Steele, 2014) and in this volume] is less obvious. At first, the reason can appear as obvious, i.e., the surveillance of health of individual cells by checking the “self” identity of cellular proteomes for the purpose of sanitation from aberrant sick, e.g., malignant and senescent, cells. The efficacy of such tissue sanitation can be achieved by precise recognition and elimination of cells presenting any kind of “non-self” proteoforms including genuine “self”, but mutant or damaged, proteins that are misfolded, misfunctioning, or toxic for the cell. Removal of such cells can be considered as a kind of selective assisted cell death involving, probably, the Toll death receptors on TK cells.

Such selective elimination of cells tagged as “non-self” *via* MHC presentation of unexpected cellular proteoforms would be part of the general molecular, cellular, and tissue-level sanitation system, eliminating bad metabolites (by specialized hydrolases), bad nucleotides in DNA (by DNA repair), bad proteins (by proteasomal degradation and lysosomal autophagy), and finally bad cells by apoptosis from within and by cellular euthanasia assisted by the immune system *via* MHC-presented antigens. The efficacy of the latter mechanism is exemplified by the immune surveillance against HNPCC MMR-deficient mutator [cells with high mutation rates, see (Taddei et al., 1997)] tumors that grow freely only when its cells acquire additional mutation in antigen-presenting MHC proteins, e.g., beta-2-microglobulin (Bicknell et al., 1996).

Presumably, mutation-bearing “self” proteins acquire an immunological “non-self” status even by single amino acid substitutions that affect protein folding and therefore the pattern of proteolytic processing for peptide (antigen) presentation by beta-2-microglobulin. Such cells are subjected to elimination by contact-activated T-killer cells. Within this scenario, immunological individualization would be positively selected to facilitate wide-range tissue sanitation by a highly sensitive and precise “non-self” discrimination. The specificity against the “non-self” would be tuned by rearrangements and sequence alterations of MHC complex according to the selective pressure shaped by the predominant diseases in particular environments—hence, the observed “ethnicity” of MHC loci (Steele, 2014).
